# Retrospective Genotyping and Whole Genome Sequencing of a Canine Parvovirus Outbreak in Bangladesh

**DOI:** 10.3390/pathogens10111373

**Published:** 2021-10-24

**Authors:** Tofazzal Md Rakib, Babu Kanti Nath, Tridip Das, Saroj Kumar Yadav, Shane R. Raidal, Shubhagata Das

**Affiliations:** 1 Department of Pathology and Parasitology, Chattogram Veterinary and Animal Sciences University, Chattogram 4225, Bangladesh; rakibtofazzal367@gmail.com; 2 School of Agriculture, Environment and Veterinary Sciences, Charles Sturt University, Wagga Wagga, NSW 2650, Australia; babukantinath@gmail.com (B.K.N.); das.vet671@gmail.com (T.D.); shraidal@csu.edu.au (S.R.R.); 3 Department of Microbiology and Veterinary Public Health, Chattogram Veterinary and Animal Sciences University, Chattogram 4225, Bangladesh; 4 Department of Medicine and Surgery, Chattogram Veterinary and Animal Sciences University, Chattogram 4225, Bangladesh; shirfraaz@gmail.com

**Keywords:** canine parvovirus, whole genome shotgun sequencing, PCR-HRM, new CPV-2a

## Abstract

Canine parvovirus 2 (CPV-2) outbreaks in close quarters such as kennels or shelters can cause substantial case fatality. Thirteen dead Labradors from a secluded kennel of security dogs presented with typical clinical signs and gross pathology of parvovirus infection. Whole genome shotgun sequencing from tissue-extracted genomic DNA detected new CPV-2a as the contributing antigenic variant. Further genotyping using polymerase chain reaction coupled with high-resolution melt assays (PCR-HRM) confirmed new CPV-2a infection in all deceased dogs. PCR-HRM of additional thirty-four clinically suspected dogs suggested that this variant is in wider community circulation, at least in the southeastern part of Bangladesh. We present complete genome sequence of the new CPV-2a variant circulating in the domestic canine population of Bangladesh.

## 1. Introduction

Canine parvovirus 2 (CPV-2) is one of the deadly viral pathogens that cause severe hemorrhagic gastroenteritis and lymphopenia in domestic dogs, particularly in young, unvaccinated puppies. Outbreaks in close quarters such as kennels or shelters may cause significant morbidity and case fatality, especially in kennels or shelters with poor housing conditions and low vaccinal coverage. Rapid diagnosis and robust genotyping are of paramount importance in such instances for clinical management of the disease as well as for epidemiological surveillance, respectively [1].

According to the most recent classification, CPV is included in the family *Parvoviridae*, subfamily *Parvovirinae*, genus *Protoparvovirus*, which forms a unique species, *Carnivore protoparvovirus 1*, along with feline parvovirus and other parvoviruses of carnivores [2]. CPV is a non-enveloped, single-stranded DNA virus that emerged in the late 1970s from a feline parvovirus (FPV)-like ancestral virus, probably via an interspecies jump to domesticated dogs [1,3]. The linear single-stranded DNA (ssDNA) genome of CPV-2 is about 5 kb in length, possess two open reading frames (ORFs) that encodes for two non-structural proteins (NS1 and NS2), and two structural proteins (VP1 and VP2) which forms the viral capsid [4,5].

CPV-2 underwent global antigenic shifts between 1978 and 1981, resulting in the emergence of a new antigenic variant CPV-2a, that regained an ability to infect cats along with other carnivores [6,7]. Three major mutations in VP2 protein at position 87 (Met to Leu), 300 (Ala to Gly), and 305 (Asp to Tyr) have driven this antigenic shift from CPV-2 to CPV-2a, expanding the host range. Thereafter, two newer variants emerged, CPV-2b in 1984 in the USA and CPV-2c in 1995 in Italy, with mutations at residue 426 (Asn in CPV-2a, Asp in CPV-2b, and Glu in CPV-2c). The latter mutation has affected the major antigenic region (epitope A) located at the top of the three-fold spike of the VP2 capsid protein [8].

More recently, newer mutants of CPV-2a/2b with the Ser297Ala have become the predominant types identified in dogs with parvoviral infection around the world and often found co-circulating with other variants [8,9,10]. The relative frequencies of circulating CPV-2 antigenic variants vary geographically and temporally, often causing sporadic outbreaks [1,8,11]. In Bangladesh, reports of canine parvovirus infection have been primarily based on clinical observations, or to some extent reliant on antigen detection kits [12,13,14]. To a lesser extent, one study claimed molecular diagnosis without any sequence validation or characterisation [13].

Next-generation sequencing (NGs) and metagenomic data mining have become the new frontier of pathogen detection in clinical diagnostic settings [15,16]. In the absence of benchmark epidemiological data, whole genome shotgun (WGS) sequencing coupled with high-throughput metagenomic pipeline can be used to identify causative pathogens with relative ease, overcoming the hurdles of expensive and sophisticated infrastructure required for systematic isolation and identification [15,17]. Recent studies have demonstrated the value of WGS-based diagnosis and genomic characterisation of parvovirus-associated diseases in different parts of the world [1,8]. Polymerase chain reaction (PCR) coupled with high-resolution melt (HRM) curve analysis provides a low-cost, rapid, and robust genotyping alternative without the need of sequencing [18]. HRM is particularly effective in determining point mutations that could specify antigenic variants when the genome is already characterised [18,19,20].

In January 2018, we investigated a parvovirus-associated disease outbreak within an elite dog squad in Chittagong, Bangladesh. Using WGS and downstream metagenomic assays, we detected and de novo-assembled the complete genome sequence of a newly emerging antigenic variant of CPV-2a with mutation Ser297Ala in VP2 protein, recognized as new CPV-2a [8,11]. Further PCR-HRM based genotype screening of clinically suspected dogs reiterated the broader community circulation of this variant, at least in the southeastern part of Bangladesh. This is the first report of complete genome sequencing and molecular genotyping of new CPV-2a from Bangladesh, which would act as an epidemiological benchmark for this rapidly evolving virus.

## 2. Results and Discussion

### 2.1. WGS and Metagenomic Investigation of CPV-2 Outbreak

**Figure 1:**
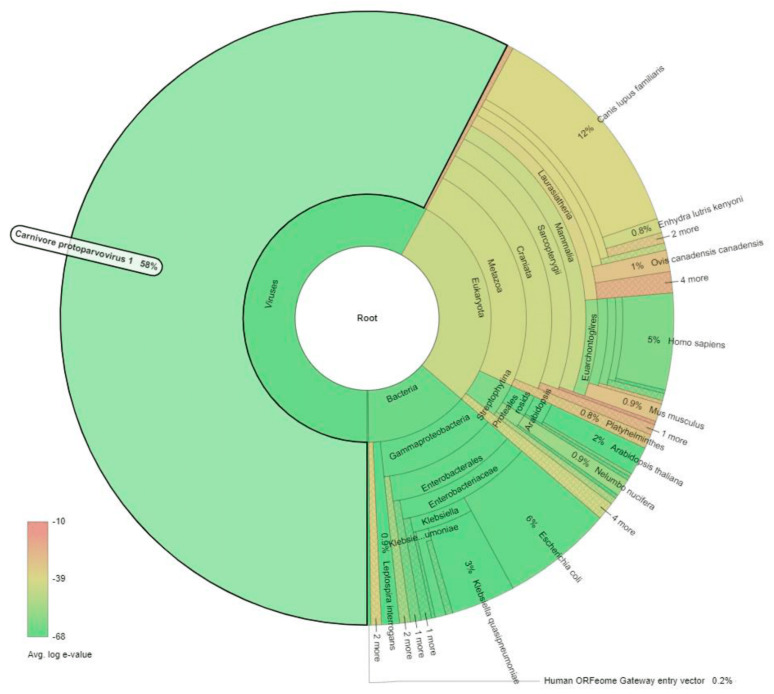
Krona chart demonstrating the relative abundance of metagenomic signals detected by MePiC screening. *Carnivore protoparvovirus 1* demonstrated the highest abundant signal with high statistical confidence (e-value), while enteric and environmental bacterial signals were also present.

Canine parvoviral enteritis has clinical similarities with other causes of acute gastrointestinal disturbances, including, but not limited to, canine distemper virus infection and other viral enteritis, hemorrhagic gastroenteritis, enteric bacterial infections, acute pancreatitis, hypoadrenocorticism, inflammatory bowel disease, intestinal intussusception, foreign body reaction, and various intoxications [21]. Thus, etiological diagnosis often requires molecular detection of viral DNA in the clinical specimen. In January 2018, within a single week timeframe, a total of thirteen dead Labrador retrievers, aged between 12 and 14 months, were presented to SAQ Teaching Veterinary Hospital (SAQTVH) of Chattogram Veterinary and Animal Sciences University, Bangladesh for postmortem examination. All deceased dogs belonged to a secluded kennel of security force and had similar clinical presentation, which included obtunded posture, acute febrile episodes, and hemorrhagic diarrhea, followed by succumbing to hypovolemic shock. Marked segmental hemorrhagic enteritis was the common gross pathological feature in all carcasses, with varying amounts of non-translucent serosanguineous fluid in the intestinal lumen, multisystemic congestion, and hemorrhage. Whole genome shotgun (WGS) sequencing of the pooled gDNA and subsequent metagenomic analysis unveiled that approximately 58% of non-host-derived reads were mapped to viruses from species *Carnivore protoparvovirus 1*, 14% from enteric bacteria under family *Enterobacteriaceae*, and the rest from Eukaryotic biota, likely ingesta and environmental material (Figure 1). WGS uses a massively parallel “sequencing by synthesis” technology which allows discovery of a vast diversity of pathogens without bias or prior knowledge of the organism involved. Coupled with established metagenomics pipelines, this approach can not only discover novel pathogens, but also provide qualitative information on the relative abundance of the pathogen [1,8]. Hence, corroborating the clinical signs, gross pathology, and paucity of other putative pathogens, CPV-2 infection was considered as the primary etiological process.

De novo assembly and subsequent reference mapping enabled reconstruction of the complete genome sequence and was deposited in GenBank under accession No. MT629886, which henceforth will be denoted as genome sequence MT629886_CVASU1. The newly assembled genome comprised 4842 bp nucleotides closely related to CPV-2a variants from China (GenBank Accession MH476593, 99.34% nucleotide identity) and India (MH545963, 99.1% nucleotide identity). The genome comprised two structural capsid protein coding ORFs, including alternatively spliced VP1 (nucleotide interval 2185–2215 and 2288–4440) and VP2 (2686–4440), as well as two overlapping nonstructural ORFs, encoding NS1 (172–2178) and NS2 (172–431) proteins.

### 2.2. Phylogenetic Reconstruction and Analysis of the Complete Genome

**Figure 2:**
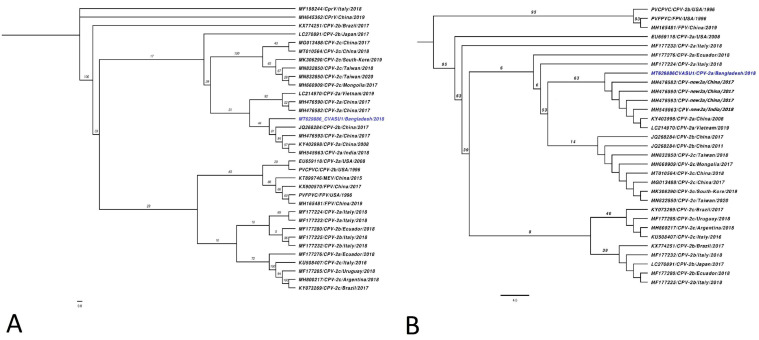
(**A**) Maximum likelihood (ML) phylogenetic inference of contemporary CPV complete genomes. The newly assembled CPV-2 sequence MT629886 (blue taxa) formed a poorly supported clade with different extant CPV-2 strains circulating in China and India. (**B**) ML tree constructed from the complete VP2 protein: the Bangladeshi CPV-2 isolate formed a statistically supported (63% bootstrap support) monophyletic clade comprised of new CPV-2a variants discovered in China. Both ML trees are rooted at the midpoint, while the proportional branch length is shown in each taxon. Genbank accession, acronym of virus variants, source country, and collection year are shown in each taxon in parentheses.

**Figure 3:**
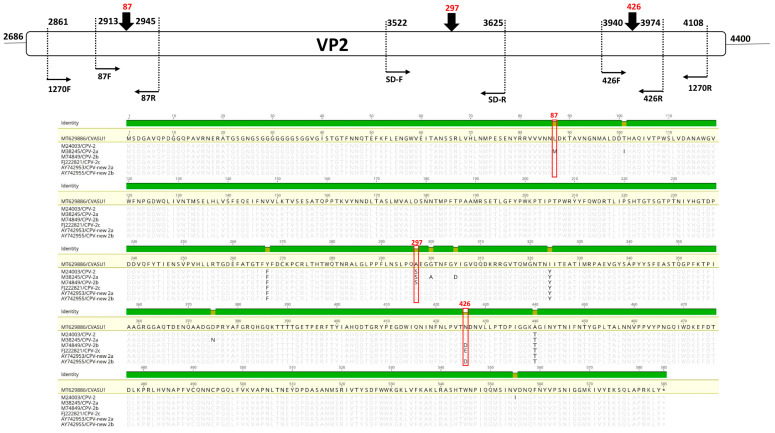
(**A**) Primer positions on the VP2 protein coding gene on CPV-2 genome used to differentiate circulating genotypes using the PCR-HRM assay. (**B**) VP2 protein alignment highlighting (red box) VP2 mutations corroborating with different CPV antigenic variants and targeted by PCR primers 87 FR, 297 CPV-SD FR, and 426 FR, respectively.

Maximum likelihood (ML) tree comprising contemporary FPV and CPV variants positioned MT629886_CVASU1 (blue taxa) within a well-supported clade of extant CPV-2a variants circulating in China (JQ268284, MH476593, KY403998) and India (MH545963) (Figure 2A). The genome was further characterised based on AA alignment of VP2 protein from all known CPV-2 variants, with subsequent phylogenetic tree reconstruction which positioned the sequence in a monophyletic clade populated with the new CPV-2a variant from China (MH476582, MH476590, MH476593, KY403998), India (MH545963), and Vietnam (LC214970) (Figure 2B). Specific mutations on AA position 87, 297, and 426 also validated this result (Figure 3B, Table S2). The major capsid protein VP2 is responsible for cellular attachment with host receptors and entry, and therefore is a major target for evolutionary divergence owing to the bottlenecks imposed by innate host defense or vaccine-induced immune response. After emerging from wild carnivores in 1978 from the last common ancestors of feline panleukopenia virus, the original CPV-2 had been replaced by an antigenic variant called CPV-2a through five/six amino acid mutations in VP2 (residues Met87Leu, Ile101Thr, Ala300Gly, Asp305Tyr, and Asn375Asp) [3,22]. Newer variants such as CPV-2b and 2c emerged as a result of selective mutation in amino acid residue 426, which enabled the virus for immune evasion and sporadic outbreak in various parts of the world [8,23]. At AA position 426, MT629886_CVASU1 had asparagine alongside specific substitutions Met87Leu, Ile101Thr, Ala300Gly, Asp305Tyr, and Asn375Asp, representing variant CPV-2a (Figure 3, Supplementary Table S2). However, substitution of Ser to Ala was also detected at AA position 297 (Figure 3B), a feature of an emerging variant known as new CPV-2a [22,24]. On the CPV capsid structure, residue 297 is a minor antigenic site close to epitope B and was speculated for changes in antigenicity [25]. In recent years, Ser297Ala substitution has been increasingly reported in CVP-2a variants of India [24,26,27], China [23], Vietnam [28], and Italy [29]. Additional Tyr324Ile and Thr440Ala substitutions might have influenced the antigenic structure due to its location in the GH loop of the VP2 protein [22].

CPV-2 antigenic variants seem to coexist in endemic populations of domestic and wild carnivores without a clear spatiotemporal pattern [30,31]. Seasonal variation of reemergence or local sporadic outbreaks of coexistent variants have been rigorously reported [32]. The latest example of this trend is the emergence of new CPV-2a, 2b, or 2c variants in China, India, and Brazil [23,33,34]. Anecdotally, these new variants might have been diverged from simple genetic drift and adaptive selection towards more virulence, and henceforth towards greater transmissibility and a broader host range [22,32]. The new CPV-2a variant has been speculated for a large-scale clinical outbreak in mainland China, Brazil, and India, with evidence of multiple vaccination failure and immune evasion [22,34,35]. This could explain the unusual succumbing of young-adults and previously vaccinated dogs observed in this study. However, the role of emerging CPV-2 variants in immunization failures is widely debated, while primary immunization failure (failure to raise protective antibody after vaccination) or secondary immunization failure (waning immunity faster than expected) may allow the vaccinated dogs to be exposed to wild-type CPV-2 infection and subsequent clinical disease. A recent review highlighted several plausible explanations of immunization failures after canine parvovirus vaccination, including vaccine-related failures such as vaccine storage or administration errors, non-compliance with vaccine schedules, and failures in vaccine immunogenicity or host-related factors such as age, genetic factors, and impaired health, nutrition, or immune status [36]. Any number of combinations of these factors might have resulted in the failure of vaccine protection in this case. In this context, one limitation of the study was that the clinical history summarized in Table S3 was reliant on the signalment form of SAQTVH, which did not record a detailed vaccination protocol or types of vaccine used. Therefore, vaccine-related failures cannot be completely ruled out as an underlying factor for the high fatality rates.

### 2.3. PCR-HRM-Based Genotyping and Investigation for Community Transmission

While our WGS approach successfully assembled the complete genome of the new CPV-2a variant in the pooled tissue specimen, it is possible that the analyses could have suffered from selection bias, ignoring concurrent infections with multiple CPV-2 variants when one genotype was serendipitously over-represented in WGS reads [37,38]. We corrected for this by implementing the PCR-HRM assay utilizing reported primer sets 87FR and 426 FR, as well as the newly designed 297 FR set targeting mutations on VP2 residue positions 87, 426, and 297, respectively (Figure 3A,B). PCR-HRM genotyping on gDNA samples of individual carcasses did not support the existence of any other concurrent antigenic variant (Figure 3A, Supplementary Figure S1a,b). This result was further validated with Sanger dideoxy sequencing, that confirmed 100% nucleotide identity of test samples with MT629886_CVASU1 (data not shown). Several studies implemented targeted amplification of the VP2 gene to identify the specific mutations that signify the different antigenic variants of CPV-2 [24,31,32]. However, this requires sequencing of the targeted amplicon and downstream bioinformatics analysis for genotyping, which is laborious, time-consuming, and costly [31]. In this context, the PCR-HRM assay provides a high-throughput detection and genotyping pipeline in a clinical setting without the need of sequencing [18]. This method implements the use of DNA intercalating dye and a sensitive algorithm to generate a characteristic melting curve that is unique for a particular genotype to the point of single nucleotide polymorphism. This is particularly applicable to identify CPV strains and antigenic variants as point mutations on VP2 protein coding genes can alter the serotype defining epitopes [20].

Similar to the rest of the word, parvoviral diarrhea was reported in the domestic dog population in Bangladesh [12,14], however prior to this study, there were no epidemiological data that included sequence analysis or variant identification. A recent report claimed molecular detection of CPV-2a, 2b, and 2c strains from rectal swabs of suspected dogs using amplification refractory mutation system (ARMS)-PCR [13], however these data were not supported by genomic sequencing or downstream genomic analyses. Hence, to obtain information on the CPV community transmission in the region, rectal swabs from 34 clinically suspected dogs were tested using the PCR-HRM approach, of which 8 were positive for CPV (23.5% of the suspected dogs) and all of them were identified as the new CPV-2a variant from melt curve data (Table S3). This result highlights that the new CPV-2a is the predominant if not the only circulating antigenic variant in the domestic dog population of the study area.

## 3. Conclusions

We investigated a fatal clinical outbreak of parvoviral disease in a previously vaccinated squad of security dogs, which serendipitously identified an emerging new CPV-2a antigenic variant. This is the first complete genome characterization of CPV circulating in Bangladesh. We also provided an example of an accelerated approach of viral outbreak investigation using WGS metagenomics and subsequent PCR-HRM-based genotyping. This approach could also be used as a pipeline for investigating emerging and remerging viral infections in clinical settings or in a low socio-economic scenario, where conventional isolation, identification, or genotyping facilities are extremely limited. Our findings suggested that the new CPV-2a might be endemic in Bangladesh and is likely the dominant antigenic variant in circulation with the potential to cause severe outbreaks. These observations underscore the importance of detailed molecular epidemiological investigation on CPV-2 in the region.

## 4. Materials and Methods

### 4.1. Samples

At necropsy, tissue samples of multiple visceral organs (liver, spleen, and intestine) were collected from all carcasses and snap frozen at −80 °C. Blood samples and rectal swabs from another thirty-four clinically suspected dogs presented at SAQTVH were also collected and stored for molecular testing.

### 4.2. Shotgun Metagenomics by Second-Generation Sequencing

#### 4.2.1. DNA Extraction and Library Construction for NGS

Genomic DNA (gDNA) was extracted from pooled tissue (intestine, spleen, and liver) samples from all carcasses, conducted using the QIAamp DNA mini kit (Qiagen, Hilden, Germany) following the manufacturer’s instructions with some modification. In brief, virion enrichment from the macerated sample was performed by centrifugation for 2 min at 8000× *g* to remove tissue debris, and the supernatants were subsequently filtered through 5 µm (Millipore) centrifuge filters [39]. The filtrates were RNase (PureLink ™ RNase A, Thermo Fisher Scientific, CA, USA) treated to remove any unprotected nucleic acids. The filtrates were used for viral DNA extraction using QIAamp DNA mini kit (Qiagen, Hilden, Germany). The extracted DNA was further sheared by ultrasound shearing using a Covaris S2 sonifier (Covaris Inc., Woburn, MA, USA) and paired-end libraries with 150 bp insert size using the Illumina paired-end sample preparation kit (Illumina, San Diego, CA, USA), according to the manufacturer’s instructions. Whole genome sequencing was performed using Illumina next-generation sequencing (NGS) technology on a HiSeq4000 platform by Annoroad Gene Technology, China.

#### 4.2.2. Metagenomic Screening from the Pooled gDNA

The raw reads (FastQ) were subjected to metagenomics screening under a cloud-computing pipeline (National Institute of Infectious Diseases, Tokyo, Japan) “MePIC: Metagenomic Pathogen Identification for Clinical specimens” [40], to detect and compare pathogen signals in the sample sets. In this pipeline, the raw sequenced reads were quality trimmed and host DNA (*Canis lupus familiaris*) was further subtracted, followed by Megablast against the in-built metagenome database (E-value 1 × 10^−10^, gap search allowed 1 hit per query, Query limit 1000), which generated 532 BLAST hits. Then, an interactive metagenomics visualization web tool (Krona, National Biodefense Analysis and Countermeasures Center, USA) was used for intuitive exploration and visualization of relative abundances and confidences within the hierarchies of metagenomic classifications [41].

#### 4.2.3. CPV Whole Genome Assembly

The sequencing with the Illumina chemistry afforded a total of 6,000,425 pairs of 150 bp reads. The raw sequencing reads were trimmed by BBDuck for removing adaptors and low-quality reads, as implemented in Geneious prime 2021.1.1. After removal of ambiguous base calls, and bases or entire reads of poor-quality using default parameters, 5,999,406 reads (99.98% of the raw reads) were used for de novo assembly. De novo assembly by SPAdes assembler v3.13.0 generated a total of 68,411 contigs. These contigs were screened against a custom BLAST database constructed based on the updated reference virus database (RVDB) version U-RBDVv21.0. MEGABLAST search on the resulted contigs confirmed the closest match with CPV-2a sequences. The selected contigs were then mapped against CPV-2a sequence as a reference genome in Geneious prime 2021.1.1 with medium sensitivity and 1000-times iteration. The mapped consensus sequence was checked carefully, and manually edited for gaps, and tweaked, ordered, and oriented where necessary. The draft genome thus generated from de novo contigs was then used as a reference genome to assemble against the trimmed reads (99.98% reads) to validate further, which matched perfectly and produced a 4842 bp consensus genome for the CPV-2a. The Geneious in-built ORF finder and protein annotation tool Glimmer v3.0 (Johns Hopkins University Center for Computational Biology, MD, USA) [42] were used to identify and annotate the canine parvovirus genes and CDs.

### 4.3. CPV Targeted PCR Amplification and HRM-Based Strain Screening

Tissue extracted gDNA from each of the diseased dogs as well as rectal swabs from 34 clinically suspected dogs were screened for CPV-2 using specific primer sets targeting VP2 genes (Table S1). PCR-positive gDNA samples were subsequently assessed with high-resolution melt curve analysis to differentiate CPV strains [19,20] based on estimating the nucleotide variation among identified CPVs. Sanger sequencing of the targeted amplicons was used to validate the genotyping where the melt temperature profile varied.

### 4.4. Phylogenetic Reconstruction

Maximum likelihood (ML) phylogenetic reconstruction was used to determine the genealogical relatedness of the newly assembled CPV-2a complete genome sequence with diverse genotypes of CPV circulating in different geographical regions of the world. For better phylogenetic resolution, complete genome sequences from closely related viruses, including *Carnivore protoparvovirus 1* (CPrV 1) and Feline panleukopenia virus (FLV), were also used. For ML tree reconstruction, selected individual sequences were annotated with accession number, antigenic variant, country of circulation, and isolate collection year. A global alignment of all full-length genomes was generated in Geneious with MAFFT v7.017 (Research Institute for Microbial diseases, Osaka, Japan) using the G-INS-i (gap open penalty 1.53; offset value 0.123) alignment algorithm [43,44] implemented in the Geneious package. For ML trees, the program jModelTest 2.1.3 favored a general-time-reversible model with gamma distribution rate variation and a proportion of invariable sites (GTR + I) for the CPV phylogeny [45]. ML trees were reconstructed using PhyML v3.3 with 100 bootstrap replicates [46]. Similarly, amino acid sequences of the complete VP2 protein from complete genomes of CPV-2 variants were annotated, aligned using default setting of Clustal omega v1.2.2, and subsequently, ML trees were reconstructed in Trex-online [47] using default parameters in PhyML 3.3 (substitution model: WAG, substitution rate categories: 4, Gamma distribution: estimated).

## Supplementary Materials

The following are available online at https://www.mdpi.com/article/10.3390/pathogens10111373/s1, Table S1: Primers used for the HRM analysis in this study, Table S2: Comparison of VP2 protein sequence with example sequences of different circulating FPV and CPV strains throughout the world, Table S3: Metadata for collected specimens in this study with PCR-HRM results. Figure S1a: HRM melt curve profile for individual PCR-positive specimens for VP2 mutations on amino acid positions 87, 297, and 426. Panels A, B, and C demonstrate the PCR-HRM melting profiles from gDNA extracted from tissue samples of the carcasses using primer sets 87 FR, 426 FR, and 297 FR, respectively. Panel D represents the PCR-HRM melt profile from PCR-positive rectal swabs using the 297 FR primer set, while two positive gDNA samples (S003165 and S003198) are used for comparison. Melt profiles of the individual samples are colored independently for visual aid, while melt T_m_ is provided on the right-hand side of each panel. In each panel, the X-axis represents temperature (°C) while the Y-axis represents the intensity of the florescence (dF/dT). Visually, there was no variation of the melting profile for the 3 target VP2 mutations (87,297 and 426); however, any variation in melt T_m_ (purple text) was further investigated using Sanger dideoxy sequencing, which revealed 100% nucleotide identity (data not shown) with other samples, which indicates that all PCR-positive dogs were infected with the same CPV-2 antigenic variant; Figure S1b: Normalized HRM melt curve profile for individual PCR-positive specimens for VP2 mutations on amino acid positions 87, 297, and 426. Panels A, B, and C demonstrate the PCR-HRM melting profiles from gDNA extracted from tissue samples of the carcasses using primer sets 87 FR, 426 FR, and 297 FR, respectively. Panel D represents the PCR-HRM melt profile from PCR-positive rectal swabs using the 297 FR primer set, while two positive gDNA samples (S003165 and S003198) are used for comparison. In each panel, the X-axis represents temperature (°C) while the Y-axis represents normalized florescence.
