# Supplementary material for: Retrospective Genotyping and Whole Genome Sequencing of a Canine Parvovirus Outbreak in Bangladesh
Source: Pathogens. 2021 Oct 24;10(11):1373. doi: 10.3390/pathogens10111373 (PMC8619975; doi:10.3390/pathogens10111373)
Supplement: Supplementary file 1 [file pathogens-10-01373-s001.zip › pathogens-1366865-supplementary.pdf]

Table S1: Primers used for the HRM analysis in this study.

Primer	Sequence	Fragment length	Interpretation	Reference
1270F	5’ TGGAAATCACAGCAAACTC 3'	1270 bp	Pre amplification and sequencing primer	(Bingga et al., 2014)
1270R	5’ AGTCTTGGTTTTAAGTCAGTATC 3'			
87F	5' TGAAAATTATAGAAGAGTGGT 3'	53 bp	CPV-2 from 2a, -2b and -2c	(Bingga et al., 2014)
87R	5' CGTTAACTGCAGTTTTATCCA 3'			
426F	5' AACTTTAACCTTCCTGTA 3'	52 bp	CPV-2a, -2b and -2c	(Bingga et al., 2014)
426R	5' TTGGATCTGTTGGTAGCA 3'			
CPV-SD-F	5' GCAAACAAATAGAGCATTGGGC 3'	124bp	CPV-new2a, new2b	This study
CPV-SD-R	5' CCCCATTTGAGTTACACCACG 3'			

Table S2: Comparison of VP2 protein sequence with example sequences of different circulating FPV and CPV strains throughout the world.

GenBank Accession number	Strain	Country	Year	Amino acid residue								
				**87**	**101**	**297**	**300**	**305**	**324**	**375**	**426**	**440**
				**Met**	**Ile**	**Ser**	**Asp**	**Asp**	**Tyr**	**Asn**	**Asn**	**Thr**
MT629886	CPV-new2a	Bangladesh	2018	Leu	Thr	Ala	Gly	Tyr	Ile	Asp	.	Ala
MH476590	CPV-new2a	China		Leu	Thr	Ala	Gly	Tyr	Ile	Asp	-	Ala
MH476593	CPV-new2a	China		Leu	Thr	Ala	Gly	Tyr	Ile	Asp	-	Ala
MH545963	CPV-new2a	India		Leu	Thr	Ala	Gly	Tyr	Ile	Asp	-	Ala
KX219733.1	CPV-New2a	India	2015	Leu	Thr	Ala	Gly	Tyr	Ile	Asp	.	Ala
M24004.1	FPV	USA	1993	.	Thr	.	Ala	.	.	Asp	.	.
M23255.1	FPV	USA	1993	.	.	.	Ala	.	.	.	.	.
M24000.1	FPV	USA	1993	Leu	Thr	.	Gly	Tyr	.	Asp	.	.
M24003.1	FPV	USA	1993	Leu	Thr	.	Gly	Tyr	.	Asp	.	.
GU569943.1	CPV 2	China	2010	Met	Ile	Ser	Asp	Asp	Tyr	Asn	-	Thr
JN625220.1	CPV-Vac	India	2012	.	.	.	Ala	.	.	.	.	.
JN625224.1	CPV-Vac	India	2012	.	.	.	.	.	.	.	.	.
FJ011097.1	CPV-Vac	Taiwan	2009	.	.	.	Ala	.	.	.	.	.
FJ011098.1	CPV-Vac	Taiwan	2009	.	.	.	Ala	.	.	.	.	.
KT162033.1	CPV-new2a	China	2014	Leu	Thr	Ala	Gly	Tyr	Ile	Asp	.	Ala
MK518017.1	CPV-new2a	China	2017	Leu	Thr	Ala	Gly	Tyr	Ile	Asp	.	Ala
DQ340404.1	CPV-2a	Brazil	1980	Leu	Thr	.	Gly	Tyr	.	Asp	.	.
DQ340410.1	CPV-2a	Brazil	1986	Leu	Thr	.	Gly	Tyr	.	Asp	.	.
DQ340415.1	CPV-new2a	Brazil	1991	Leu	Thr	Ala	Gly	Tyr	.	Asp	.	.

DQ340430.1	CPV- new2a	Brazil	1995	Leu	Thr	Asn	Gly	Tyr	.	Asp	.	.
DQ340434.1	CPV- new2a	Brazil	2000	Leu	Thr	Ala	Gly	Tyr	.	Asp	.	.
GU569941.1	CPV- new2a	China	2002	Leu	Thr	Ala	Gly	Tyr	.	.	.	.
GU380298.1	CPV- new2a	China	2009	Leu	Thr	Ala	Gly	Tyr	Ile	Asp	.	Ala
JN867597.1	CPV- new2a	USA	2010	Leu	Thr	Ala	.	His	.	Asp	.	.
KX219736.1	CPV- new2a	India	2012	Leu	Thr	Ala	Gly	Tyr	Ile	Asp	.	Ala
KR002805.1	CPV- new2a	China	2014	Leu	Thr	Ala	Gly	Tyr	Ile	Asp	.	Ala
KM386821.1	CPV- new2a	China	2014	Leu	Thr	Ala	Gly	Tyr	Ile	Asp	.	.
KR559896.1	CPV- new2a	Portugal	2014	Leu	Thr	Ala	Gly	Tyr	.	Asp	.	.
KX618915.1	CPV- new2a	Singapore	2017	Leu	Thr	Ala	.	Tyr	Ile	Asp	.	Ala
MN053892.1	CPV- new2a	South Korea	2017	Leu	Thr	Ala	Gly	Tyr	Ile	Asp	.	Ala
MH545963.1	CPV- new2a	India	2018	Leu	Thr	Ala	Gly	Tyr	Ile	Asp	.	Ala
MK895484.1	CPV- new2a	Nigeria	2018	Leu	Thr	Ala	Gly	Tyr	Ile	Asp	.	Ala
DQ340409.1	CPV-2b	Brazil	1985	Leu	Thr	.	Gly	Tyr	.	Asp	Asp	.
FJ005260.1	CPV- new2b	Germany	1997	Leu	Thr	Ala	Gly	Tyr	.	Asp	Asp	.
M38245.1	CPV-2b	USA	1996	.	.	.	Ala	.	.	.	.	.
MF177225.1	CPV- new2b	Italy	1998	Leu	Thr	Ala	Gly	Tyr	.	Asp	Asp	.
GU569944.1	CPV- new2b	China	2002	Leu	Thr	Ala	Gly	Tyr	.	.	Asp	.
JF414817.1	CPV-2b	Argentina	2003	Leu	Thr	Asn	Gly	Tyr	.	Asp	Asp	.
GU212792.1	CPV-2b	Thailand	2009	.	.	.	Ala	.	.	Glu	Asp	.
KX425921.1	CPV- new2b	India	2010	Leu	Thr	Ala	Gly	Tyr	.	Asp	Asp	.
JQ743893.1	CPV- new2b	China	2011	Leu	Thr	Ala	Gly	Tyr	Ile	Asp	Asp	.
KX469432.1	CPV- new2b	India	2011	Leu	Thr	Ala	Gly	Tyr	.	Asp	Asp	.

KR559892.1	CPV- new2b	Portugal	2012	Leu	Thr	Ala	Gly	Tyr	.	Asp	Asp	.
KP682515.1	CPV- new2b	Spain	2013	Leu	Thr	Ala	Gly	Tyr	.	Asp	Asp	.
KR559895.1	CPV- new2b	Portugal	2013	Leu	Thr	Ala	Gly	Tyr	.	Asp	Asp	.
MF177258.1	CPV-2b	Brazil	2013	Leu	Thr	Asn	Gly	Tyr	Leu	Asp	Asp	.
KX774249.1	CPV- new2b	Brazil	2014	Leu	Thr	Ala	Gly	Tyr	Leu	Asp	Asp	.
KX774251.1	CPV- new2b	Brazil	2015	Leu	Thr	Ala	Gly	Tyr	Leu	Asp	Asp	.
KX774252.1	CPV- new2b	Brazil	2015	Leu	Thr	Ala	Gly	Tyr	Leu	Asp	Asp	.
MN053880.1	CPV- new2b	South Korea	2016	Leu	Thr	Ala	Gly	Tyr	Ile	Asp	Asp	Ala
LC270891.1	CPV- new2b	Japan	2017	Leu	Thr	Ala	Gly	Tyr	.	Asp	Asp	.
LC270892.1	CPV- new2b	Japan	2017	Leu	Thr	Ala	Val	Tyr	.	Asp	Asp	.
FJ005198.1	CPV-2c	Germany	1997	Leu	Thr	Ala	Gly	Tyr	.	Asp	Glu	.
MF177227.1	CPV-2c	France	1997	Leu	Thr	Ala	Gly	Tyr	.	Asp	Glu	.
FJ222824.1	CPV-2c	Italy	2005	.	.	.	Ala	.	.	.	.	.
FJ005235.1	CPV-2c	USA	2007	Leu	Thr	Ala	Gly	Tyr	.	Asp	Glu	.
MF177284.1	CPV-2c	Uruguay	2009	Leu	Thr	Ala	Gly	Tyr	.	Asp	Glu	Ala
KF482468.1	CPV-2c	China	2009	Leu	Thr	Ala	Gly	Tyr	.	Asp	Asp	.
GU380303.1	CPV 2c	China	2010	Leu	Thr	Ala	Gly	Tyr	Ile	Asp	Glu	.
KX425920.1	CPV-2c	India	2010	Leu	Thr	Ala	Gly	Tyr	.	Asp	Glu	.
KR559894.1	CPV-2c	Portugal	2012	Leu	Thr	Ala	Gly	Tyr	.	Asp	Glu	.
KT275253.1	CPV-2c	Portugal	2012	Leu	Thr	Ala	Gly	Tyr	.	Asp	Glu	.
LC216910.1	CPV-2c	Indonesia	2013	Leu	Thr	Ala	Gly	Tyr	Ile	Asp	Glu	.
LC214969.1	CPV-2c	Viet Nam	2013	Leu	Thr	Ala	Gly	Tyr	Ile	Asp	Glu	.
MH800217.1	CPV-2c	Argentina	2013	Leu	Thr	Ala	Gly	Tyr	.	Asp	Glu	.

KT162019.1	CPV-2c	China	2014	Leu	Thr	Ala	Gly	Tyr	Ile	Asp	Glu	.
KP859578.1	CPV-2c	Croatia	2014	Leu	Thr	Ala	Gly	Tyr	.	Asp	Glu	.
KU244254.1	CPV-2c	Taiwan	2015	Leu	Thr	Ala	Gly	Tyr	Ile	Asp	Glu	.
KU508692.1	CPV-2c	Australia	2015	Leu	Thr	Ala	Gly	Tyr	.	Asp	Glu	.
MG264077.1	CPV-2c	Ecuador	2017	Leu	Thr	Ala	Gly	Tyr	.	Asp	Glu	.
MH660909.1	CPV-2c	Mongolia	2017	Leu	Thr	Ala	Gly	Tyr	Ile	Asp	Glu	.
MK268682.1	CPV-2c	China	2018	Leu	Thr	Ala	Gly	Tyr	Ile	Asp	Glu	.

NB: '**.**' Indicates the presence of same amino acid residue as stated in the heading

Table S3: Metadata for collected specimen in this study with PCR-HRM results.

SL No.	Specimen Voucher	Breed	Age	Sex	Sample	Sample Date	Location	Vaccination	PCR-HRM
1	S003165	Labrador	14 m	M	Tissue	2018/01/13	22.06525330330806, 92.27824118601515	Yes	Pos
2	S003198	Labrador	16 m	M	Tissue	2018/01/13	22.06525330330806, 92.27824118601515	Yes	Pos
3	S003277	Labrador	16 m	M	Tissue	2018/01/13	22.06525330330806, 92.27824118601515	Yes	Pos
4	S003504	Labrador	12 m	M	Tissue	2018/01/14	22.06525330330806, 92.27824118601515	Yes	Pos
5	S003506	Labrador	14 m	F	Tissue	2018/01/14	22.06525330330806, 92.27824118601515	Yes	Pos
6	S003713	Labrador	14 m	M	Tissue	2018/01/14	22.06525330330806, 92.27824118601515	Yes	Pos
7	S003714	Labrador	14 m	M	Tissue	2018/01/14	22.06525330330806, 92.27824118601515	Yes	Pos
8	S003757	Labrador	15 m	M	Tissue	2018/01/14	22.06525330330806, 92.27824118601515	Yes	Pos
9	S003891	Labrador	15 m	M	Tissue	2018/01/16	22.06525330330806, 92.27824118601515	Yes	Pos
10	S003900	Labrador	13 m	M	Tissue	2018/01/17	22.06525330330806, 92.27824118601515	Yes	Pos
11	S003917	Labrador	14 m	M	Tissue	2018/01/17	22.06525330330806, 92.27824118601515	Yes	Pos
12	S004003	Labrador	12 m	F	Tissue	2018/01/17	22.06525330330806, 92.27824118601515	Yes	Pos
13	S004319	Labrador	14 m	M	Tissue	2018/01/18	22.06525330330806, 92.27824118601515	Yes	Pos
14	S003698	German Shephard	11 m	F	Rectal Swab	2018/02/22	22.36042042029397, 91.79353601968192	Yes	Neg
15	S003169	German Shephard	6 m	M	Rectal Swab	2018/02/22	22.36042042029397, 91.79353601968192	Yes	Pos

16	S003654	German Shephard	16 m	F	Rectal Swab	2018/02/22	22.36042042029397, 91.79353601968192	Unknown	Pos
17	S003891	German Shephard	21 m	M	Rectal Swab	2018/02/28	22.36042042029397, 91.79353601968192	Yes	Neg
18	S003986	German Shephard	30 m	M	Rectal Swab	2018/02/28	22.36042042029397, 91.79353601968192	Yes	Neg
19	S003426	German Shephard	7 m	M	Rectal Swab	2018/03/01	22.36042042029397, 91.79353601968192	Yes	Neg
20	S003687	German Shephard	13 m	M	Rectal Swab	2018/03/01	22.36042042029397, 91.79353601968192	Yes	Neg
21	S003879	German Shephard	45 d	M	Rectal Swab	2018/03/04	22.36042042029397, 91.79353601968192	No	Neg
22	S002978	German Shephard	60 d	M	Rectal Swab	2018/03/04	22.36042042029397, 91.79353601968192	No	Neg
23	S002789	German Shephard	8m	F	Rectal Swab	2018/03/04	22.36042042029397, 91.79353601968192	No	Pos
24	S003549	German Shephard	24 m	M	Rectal Swab	2018/03/05	22.36042042029397, 91.79353601968192	Yes	Neg
25	S002769	German Shephard	19 m	M	Rectal Swab	2018/03/19	22.36042042029397, 91.79353601968192	Yes	Pos
26	S003286	German Shephard	3 m	M	Rectal Swab	2018/03/19	22.36042042029397, 91.79353601968192	Yes	Neg
27	S003346	German Shephard	21 m	M	Rectal Swab	2018/03/19	22.36042042029397, 91.79353601968192	Yes	Pos
28	S003569	German Shephard	11 m	M	Rectal Swab	2018/03/19	22.36042042029397, 91.79353601968192	Yes	Neg
29	S002489	German Shephard	33 m	F	Rectal Swab	2018/03/20	22.36042042029397, 91.79353601968192	Yes	Neg
30	S002697	Labrador	6 m	F	Rectal Swab	2018/03/20	22.36042042029397, 91.79353601968192	Yes	Neg
31	S002365	Labrador	2 yrs	M	Rectal Swab	2018/03/20	22.36042042029397, 91.79353601968192	Yes	Neg
32	S002148	German Shephard	2 yrs	M	Rectal Swab	2018/03/21	22.36042042029397, 91.79353601968192	Yes	Neg

33	S002871	Local nondescriptive	3 yrs	F	Rectal Swab	2018/03/21	22.36042042029397, 91.79353601968192	No	Neg
34	S003796	Spitz	2 yrs	M	Rectal Swab	2018/03/22	22.36042042029397, 91.79353601968192	Yes	Neg
35	S003797	Spitz	13 m	M	Rectal Swab	2018/03/22	22.36042042029397, 91.79353601968192	Yes	Neg
36	S003815	German Shephard	12 m	F	Rectal Swab	2018/03/25	22.36042042029397, 91.79353601968192	Yes	Neg
37	S003145	Local nondescriptive	3 yrs	F	Rectal Swab	2018/03/25	22.36042042029397, 91.79353601968192	No	Neg
38	S002896	Labrador	18 m	F	Rectal Swab	2018/03/25	22.36042042029397, 91.79353601968192	Yes	Neg
39	S002965	Local nondescriptive	2 yrs	M	Rectal Swab	2018/03/25	22.36042042029397, 91.79353601968192	Unknown	Pos
40	S002966	Local nondescriptive	2 yrs	M	Rectal Swab	2018/03/25	22.36042042029397, 91.79353601968192	No	Pos
41	S002967	Local nondescriptive	6 m	M	Rectal Swab	2018/03/25	22.36042042029397, 91.79353601968192	Unknown	Pos
42	S003789	Spitz	12 m	F	Rectal Swab	2018/03/26	22.36042042029397, 91.79353601968192	Yes	Neg
43	S003790	Spitz	18 m	M	Rectal Swab	2018/03/26	22.36042042029397, 91.79353601968192	Yes	Neg
44	S002961	Local nondescriptive	12 m	M	Rectal Swab	2018/03/29	22.36042042029397, 91.79353601968192	No	Neg
45	S003587	Labrador	2 yrs	M	Rectal Swab	2018/03/29	22.36042042029397, 91.79353601968192	Yes	Neg
46	S003198	Spitz	3 yrs	F	Rectal Swab	2018/03/29	22.36042042029397, 91.79353601968192	Yes	Neg
47	S002839	Local nondescriptive	21 m	M	Rectal Swab	2018/03/29	22.36042042029397, 91.79353601968192	No	Neg

m=month(s); yrs= years; M=male; F=female, Pos=positive; Neg=Negative; Tissue: Intestinal mucosa, spleen, liver

Figure S1a: HRM melt curve profile for individual PCR positive specimen for VP2 mutations on amino acid position 87, 297 and 426. Panel A, B and C demonstrates PCR-HRM melting profile from gDNA extracted from tissue samples of the dead carcasses using primer sets 87FR, 426FR and 297FR respectively. Panel D represents PCR-HRM melt profile from PCR positive rectal swabs using 297FR primer set while two positive gDNA samples (S003165 and S003198) used for comparison. Melt profile of the individual samples colored independently for visual aid while melt Tm provided on the right hard side of each panel. In each panel, X-axis represents temperature (ºC) while Y axis represent intensity of the florescence (dF/dT). Visually there was no variation of melting profile for the 3 target VP2 mutations (87,297 and 426); however any variation in melt Tm (purple texts) was further investigated using Sanger dideoxy sequencing while revealed 100% nucleotide identity (data not shown) with other samples which indicate all PCR positive dogs were infected with same CPV-2 antigenic variant.

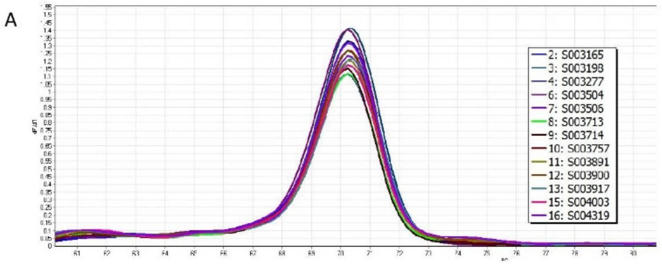

Sample ID	Melt Tm
S003165	70.25
S003198	70.35
S003277	70.25
S003504	70.25
S003506	70.25
S003713	70.25
S003714	70.25
S003757	70.25
S003891	70.25
S003900	70.25
S003917	70.4
S004003	70.25
S004319	70.25

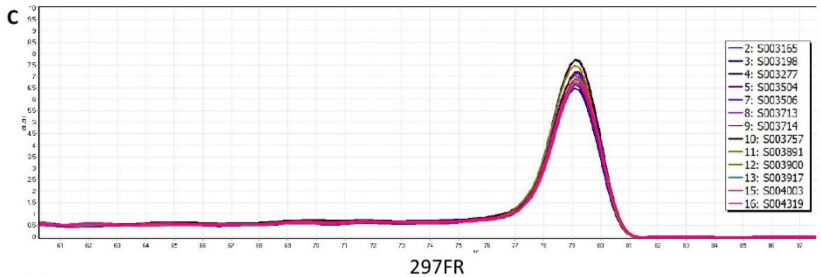

Sample ID	Melt Tm
S003165	79.1
S003198	79.15
S003277	79.1
S003504	79.1
S003506	79.1
S003713	79.15
S003714	79.15
S003757	79.15
S003891	79.1
S003900	79.15
S003917	79.15
S004003	79.15
S004319	79.15

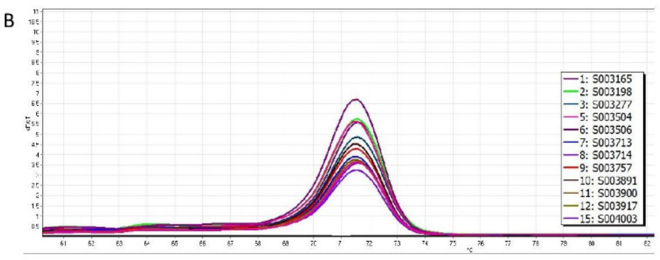

Sample ID	Melt Tm
S003165	71.6
S003198	71.5
S003277	71.6
S003504	71.5
S003506	71.5
S003713	71.5
S003714	71.5
S003757	71.5
S003891	71.5
S003900	71.5
S003917	71.5
S004003	71.6
S004319	71.6

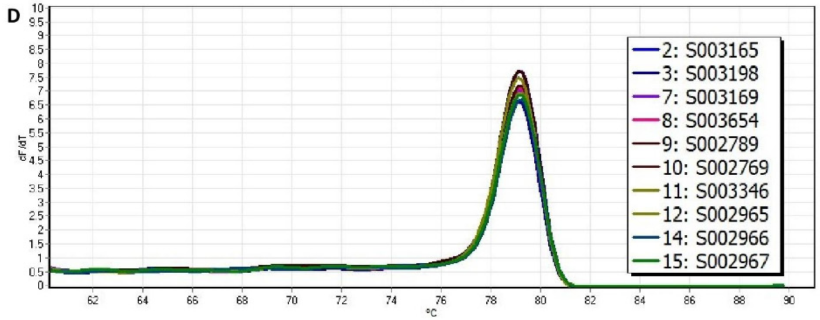

Sample ID	Melt Tm
**S003165**	**79.15**
**S003198**	**79.1**
S003169	79.15
S003654	79.15
S002789	79.15
S002769	79.15
S003346	79.15
S002965	79.15
S002966	79.20
S002967	79.15

Figure S1b: Normalized HRM melt curve profile for individual PCR positive specimen for VP2 mutations on amino acid position 87, 297 and 426. Panel A, B and C demonstrates PCR-HRM melting profile from gDNA extracted from tissue samples of the dead carcasses using primer sets 87FR, 426FR and 297FR respectively. Panel D represents PCR-HRM melt profile from PCR positive rectal swabs using 297FR primer set while two positive gDNA samples (S003165 and S003198) used for comparison. In each panel, X-axis represents temperature (ºC) while Y axis represent normalized florescence.

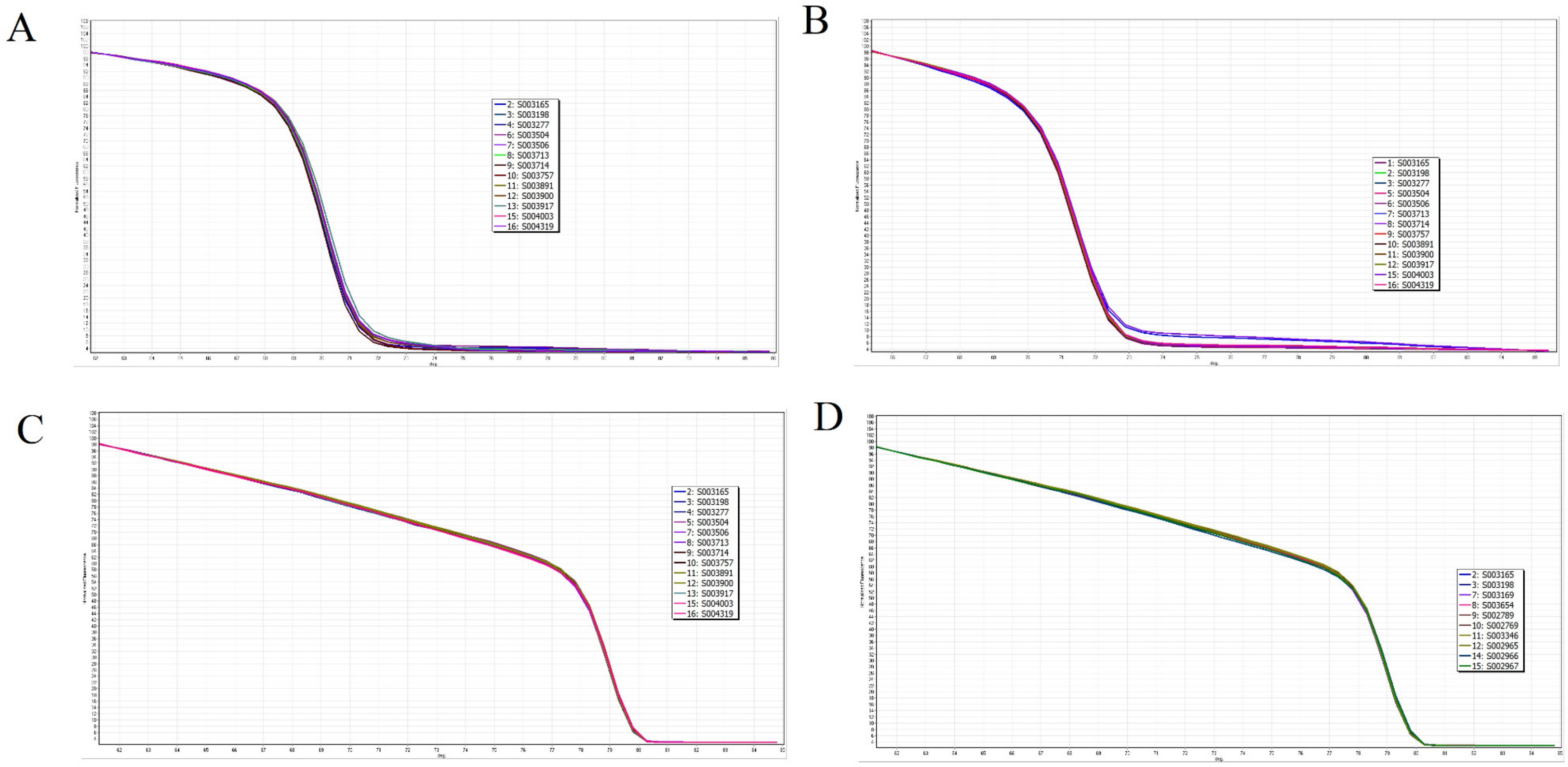

Reference:

Bingga, G., Liu, Z., Zhang, J., Zhu, Y., Lin, L., Ding, S., Guo, P., 2014. High resolution melting curve analysis as a new tool for rapid identification of canine parvovirus type 2 strains. Molecular and cellular probes 28, 271-278.
